# Evaluating an AI-Enabled Mobile Mental Health Monitoring Tool Among Family Caregivers of Adults Living With Cancer: Single-Arm Feasibility and Acceptability Trial Protocol

**DOI:** 10.2196/83276

**Published:** 2026-02-12

**Authors:** Chiara Acquati, Michael Aratow, Tahmida Nazreen, Arunima Bhattacharjee, Isabella K Marra, Ashley S Alexander

**Affiliations:** 1 Graduate College of Social Work University of Houston Houston, TX United States; 2 Department of Family Medicine and Community Health University of Minnesota Minneapolis, MN United States; 3 Ellipsis Health San Francisco, CA United States; 4 Fertitta Family College of Medicine University of Houston Houston, TX United States; 5 Kelsey Research Foundation Houston, TX United States

**Keywords:** cancer, caregiving, psychological distress, speech-based monitoring, artificial intelligence (AI), feasibility & acceptability study, mobile health (mHealth), acoustic analysis, semantic analysis, depression, anxiety

## Abstract

**Background:**

Psychological distress, particularly symptoms of depression and anxiety (D&A), is highly prevalent among family caregivers of individuals living with cancer, who often assume central roles in care coordination, treatment adherence, symptom monitoring, and emotional support. Rates of distress among caregivers frequently equal or exceed those observed in patients themselves. Despite increased attention to caregivers’ mental health needs, routine distress screening remains limited in oncology care settings. Advances in mobile health technology and artificial intelligence (AI) offer opportunities to address these needs by providing accessible and user-driven tools. The Ellipsis Caregiver Assessment Enhancement (eCARE; Ellipsis Health, Inc) is a speech-based, AI-enabled mobile app designed to screen and monitor symptoms of depression and anxiety. By collecting brief voice recordings and in-app survey data, eCARE offers a scalable approach for integrating caregiver distress monitoring into cancer care.

**Objective:**

This single-arm trial will evaluate the feasibility and acceptability of the eCARE app among family members who are the primary caregivers of patients diagnosed with cancer within the past 5 years. Specifically, the study aims to (1) determine feasibility based on platform completion rates, (2) assess acceptability using validated measures, and (3) identify barriers and facilitators influencing the uptake and sustained use of eCARE.

**Methods:**

In Phase 1, a total of 60 United States–based family caregivers will be recruited from community health clinics, cancer and caregiving advocacy groups, and online postings. Screened and enrolled caregivers will complete 6 eCARE sessions over an 8-week period. Pre- and posttest surveys assess depression, anxiety, caregiving burden, and relational processes. Feasibility will be evaluated based on the proportion of participants who complete at least 66% of weekly assessments, and acceptability will be assessed using the acceptability of intervention measure (AIM). In Phase 2, a total of 20 caregivers will be invited to participate in semi-structured online interviews to explore user experience, including perceived benefits, barriers to use, and preferences for future implementation. Qualitative data will be analyzed thematically to inform tool refinement.

**Results:**

The study has received Institutional Review Board approval from the University of Houston. Participant recruitment and enrollment began in June 2024, with data collection expected to conclude by August 2025. Data analysis will begin in December 2025, with preliminary results anticipated by May 2026.

**Conclusions:**

This study will generate preliminary evidence on the feasibility, acceptability, and utility of a speech-based, AI-enabled smartphone tool for monitoring D&A symptoms among family cancer caregivers. Findings will inform the design of a larger, fully powered trial and guide future implementation of remote psychological distress monitoring strategies in oncology care. By offering a low-burden, caregiver-centered approach, eCARE has the potential to expand access to psychosocial support and facilitate timely identification of needs and coordination of services across cancer care settings.

**International Registered Report Identifier (IRRID):**

DERR1-10.2196/83276

## Introduction

### Background

A cancer diagnosis is often associated with psychological challenges affecting patients and the broader support network, including spouses, family members, and friends who assume the role of caregivers [1-4]. Caregiving entails practical and supportive tasks across the continuum of care: coordinating medical visits, building relationships with clinicians, communicating with the medical team, and contributing to decision-making, adherence to treatment plans, and symptom management [2,3]. These tasks carry physical, psychosocial, emotional, and financial consequences, rendering cancer caregivers highly vulnerable to stress and mental health problems [3,5,6]. Across studies, family caregivers of patients with cancer often experience greater psychological distress than patients [2,4,7-11], and cancer caregiving is rated as more burdensome than other high-intensity caregiving scenarios [12]. Yet, caregivers’ mental health needs remain insufficiently addressed in routine cancer care, underscoring the need for targeted psychosocial interventions [13,14].

Caregiver psychological distress is common throughout the illness trajectory, with patterns that differ across diagnosis, active treatment, recurrence, and advanced disease. Between 55% and 95% of caregivers [15-17] report clinically significant distress or mental health conditions. Compared with the general US population, family caregivers of patients with advanced cancer frequently score below 30% on mental health measures [18]. Inadequate preparation for the caregiving role compounds distress, contributing to feelings of helplessness and regret as caregivers witness symptom burden [19,20]. Specific conditions are highly prevalent. Depression ranges from 16% to 67% and tends to increase as prognosis worsens [9,21-24]. Anxiety is even more common; a global meta-analysis estimates 46.5% of family caregivers experience clinically significant anxiety, with rates in advanced disease (~40%-42%) exceeding those observed in patients [8,22,25,26]. In some cancer types (eg, head and neck) up to 57% of caregivers report clinically significant anxiety and/or depression [27].

Multiple factors contribute to this heightened vulnerability. Caregiver risk factors include being female, younger age, financial strain, limited social support, high burden, low caregiving self-efficacy, and prolonged caregiving hours [22,24]. Family caregivers are more likely to experience psychological distress when the patient is younger, when the illness progresses and worsens, when the patient has limited awareness of their prognosis, or when the patient’s overall health and daily functioning decline [8,22,24]. Relational and contextual risk factors include being a spousal caregiver, insecure attachment (eg, fear of abandonment or avoidance), and family conflicts, which amplify distress and cause psychological strain among family cancer caregivers [21,22,24].

Despite this evidence, caregiver support remains underresourced and inadequate. Family caregivers frequently report unmet informational and psychological needs [4,11,26,28-31]. Traditional in-person mental health services are often inaccessible due to cost, time, and logistical barriers, limiting opportunities to address caregivers’ needs [1]. As a result, family caregivers experience sleep disruption, diminished quality of life, and reduced caregiving capacity, ultimately affecting patient care [1,12,32].

Digital mental health offers a scalable path to support cancer caregivers. Smartphone- and web-based interventions can extend reach, reduce access barriers, and deliver timely, evidence-based care. However, rigorous evaluation in oncology caregiving remains limited. To date, eHealth interventions for family caregivers of individuals living with cancer have been found to reduce depression and modestly improve quality of life versus usual care, supporting the feasibility and potential value of remote delivery for caregiver mental health. Still, effects were small and trials were few, with short follow-up and heterogeneous content, therefore underscoring the need for larger, higher-quality randomized controlled trials [1,6,33-37]. Building on this foundation, AI-enabled depression and anxiety (D&A) screening and monitoring apps have strong potential for flexible, real-time support [34,36,37]. Evidence shows that technology-based approaches can (1) improve health and psychological outcomes, (2) expand access to care, (3) be cost-effective, and (4) be delivered in self-paced, tailored formats [35,38-41]. Yet, family members remain excluded from national distress screening mandates, leaving their needs inconsistently identified and addressed in oncology care settings [42-45]. This evidence emphasizes an urgent need to design, test, and implement family caregiver-specific digital psychosocial interventions that can be integrated into routine cancer care.

### Advancements and Gaps in Speech-Based Mental Health Assessment

Technological advancements in automated and AI-enabled speech analysis have significantly improved the ability to detect psychological symptoms [46-49]. Tools using voice data, particularly acoustic features, now offer a promising complement to traditional validated screening instruments. These tools have shown diagnostic performance comparable to widely used psychometric scales [50,51] with added advantages including reduced user burden, minimal risk of human error (eg, missing data), and the potential for greater user comfort during verbal self-expression [52-54].

Nevertheless, several limitations persist. Most tools continue to rely on either acoustic (ie, how something is said) or semantic content (ie, what is said), without combining both for improved precision [49,55-57]. Additionally, many systems have focused solely on depression, with a smaller fraction targeting anxiety and even fewer addressing both concurrently [55-57]. This gap is particularly concerning for family caregivers of patients with cancer, a population in which anxiety symptoms are both prevalent and frequently co-occur with depressive symptomatology.

This study addresses these gaps by evaluating Ellipsis Caregiver Assessment Enhancement (eCARE; Ellipsis Health, Inc), a next-generation speech-based assessment platform designed to detect both D&A symptom severity using an integrated AI model. Unlike most existing systems, eCARE combines semantic and acoustic data inputs to provide dual assessments, enhancing precision. Additionally, it offers a clinician-facing dashboard to support referrals and care coordination, promoting a person-centered model of supportive care delivery. The Ellipsis Health platform has been technically validated in multiple peer-reviewed publications demonstrating its strong machine learning performance [58-62].

### eCARE App

eCARE is a speech-based smartphone app linked to Ellipsis Health, which is a secure, cloud-based AI infrastructure. The app processes a weekly 90-second audio recording from users who respond to prompted topics, including those related to their caregiving stress and emotional well-being. The system analyzes both the acoustic and semantic characteristics of speech using advanced deep learning techniques, including transfer learning. This approach allows for more sophisticated and accurate identification of users’ speech signals than traditional models that rely on fixed acoustic features such as pitch or volume [57,60,62].

The platform has demonstrated strong performance metrics, with reported area under the curve values of 0.85 for Patient Health Questionnaire-9 item (PHQ-9) and 0.84 for Generalized Anxiety Disorder-7 item (GAD-7) predictions (binary classification with a cutoff score of 10), root-mean-square error values of 4.25 and 4.47, and mean absolute error values of 3.13 and 3.23, respectively [63]. To date, eCARE remains the only available speech-based screening platform capable of producing concurrent D&A scores for family caregivers of patients with cancer. This dual capability is a critical feature for reducing participant fatigue during long-term monitoring and managing the acute mental health needs of this group.

The eCARE app is available for download on the Apple or the Google Play Store and can be used on iOS and Android mobile devices. After downloading the app on their smartphones, users are able to register in the app with the phone number they provided to the research team. Once a week, users will be prompted to log in to the app and record a 90-second response to a selected topic related to their mental health or caregiving responsibilities. Afterward, users will receive nonclinical feedback on their distress levels. The app also includes longitudinal tracking of responses and mental health crisis hotline information. On the provider side, eCARE features a HIPAA (Health Insurance Portability and Accountability Act)-compliant web portal that visualizes severity of distress trajectories over time, supporting timely mental health referrals and opportunities for direct integration into the cancer care continuum.

### Specific Aims

The present study will (1) expand understanding of the psychosocial issues faced by family caregivers of individuals diagnosed with cancer and (2) generate actionable evidence to refine eCARE for responsive delivery in a way that is receptive to preferences for care, format, and optimal timing of screening. Specific Aim 1: establish the feasibility and acceptability of eCARE among family caregivers of patients with cancer in a single-arm prospective cohort study, using platform completion rate (feasibility) and the acceptability of the intervention measure (AIM; acceptability) as indicators. Specific Aim 2: qualitatively evaluate facilitators and barriers to eCARE uptake among a subset of participants (n=20) to inform iterative refinement. Expected outcomes include evidence that a caregiver-driven digital approach can increase the proportion of family caregivers who are screened and monitored, as well as generating concrete recommendations to optimize eCARE for future efficacy testing and implementation in survivorship care.

## Methods

### Study Design

This single-arm, prospective cohort study will use a mixed methods design to evaluate the feasibility and acceptability of eCARE, a digital AI-enabled tool designed to monitor psychological distress among family caregivers of individuals living with cancer. The study will:

1. Track psychological distress, specifically D&A over an 8-week period among a cohort of 60 family cancer caregivers;
2. Assess adherence to and perceived acceptability of the eCARE tool;
3. Conduct semi-structured qualitative interviews with a subsample of participants (n=20) to explore experiences with eCARE use.

Participants will use eCARE independently, following structured onboarding and usage instructions provided by the research team at baseline. Psychological distress will be monitored using standardized questionnaires and voice-based data collection, with app-based metrics and quantitative survey data supplemented by qualitative feedback (Table 1).

**Table 1:** Study measures and assessment time points.

Measures	Measure administration time points and personnel			
	T0/Baseline	T1-T6	T7	Administration
Depression (PHQ-9^a^) [64]	✓	✓	✓	Qualtrics/ platform data^b^
Anxiety (GAD-7^c^) [65]	✓	✓	✓	Qualtrics/ platform data
Quality of Life (CDC 4 items^d^) [66]	✓		✓	Qualtrics
Caregiving Burden (Short Form Zarit Burden Interview) [67]	✓		✓	Qualtrics
Closeness (Inclusion of the Other in the Self Scale) [68,69]	✓		✓	Qualtrics
Communication (Social Constraints Scale) [70,71]	✓		✓	Qualtrics
Responsiveness (Perceived Partner Responsiveness Scale) [72,73]	✓		✓	Qualtrics
Communal Motivation to Care (Partner-Specific Communal Motivation Scale) [74,75]	✓		✓	Qualtrics
eCARE^e^ App Use		✓		Platform data
Acceptability of intervention measure (AIM) [76]			✓	Qualtrics
User Engagement Scale–Short Form [77]			✓	Qualtrics
Qualitative interviews			✓	RS^f^ via Zoom

^a^PHQ-9: Patient Health Questionnaire-9 item.

^b^Platform data: eCARE data collection.

^c^GAD-7: Generalized Anxiety Disorder-7 item.

^d^CDC 4 items: Centers for Disease Control and Prevention 4-item Healthy Days Measure.

^e^eCARE: Ellipsis Caregiver Assessment Enhancement.

^f^RS: research staff.

### Participant Eligibility

Participants will be eligible to enroll if they (1) self-identify as the primary caregiver or support person of an individual diagnosed with cancer within the past 5 years, (2) are 18 years of age or older, (3) have access to a smartphone capable of downloading and using the eCARE app, (4) are fluent in English, and (5) are able and willing to provide informed consent. To ensure adequate representation of caregiving experiences, a stratified sampling approach will be used to recruit equal numbers of caregivers who identify as non-Hispanic White (n=15), Black/African American (n=15), Hispanic and Latino (n=15), and Asian American, Native Hawaiian, and Pacific Islander (AANHPI; n=15). Participants will be excluded from the study if they (1) have a cognitive impairment; (2) have a speech impairment; and (3) have a severe mental illness that would impede the ability to provide informed consent or complete study activities. Eligibility will be self-reported through an initial study information survey. Participants will review a list of exclusionary criteria and be asked to self-select out of the study if any listed conditions apply. Eligibility will be verified as necessary via follow-up communication (eg, telephone screening). To minimize fraudulent responses, we will apply minimum time-on-task thresholds, checks for duplicate IPs or devices, and manual pattern review.

### Participant Recruitment and Study Procedures

#### Recruitment Procedures

Recruitment efforts will be conducted in collaboration with the Kelsey Research Foundation and other community partners. A recruitment email will be distributed directly to potential participants via the Kelsey Research Foundation listserv, support group lists, and through oncology social workers. Potential participants may self-refer to the study in one of the following ways: (1) by accessing the study flyer’s URL or scanning the QR code, which will link to an online eligibility screening survey containing study information; (2) by contacting study staff, who will provide additional information and instructions to complete the screening survey; and (3) by visiting the study landing page on the Kelsey Research Foundation website and completing a brief informational survey. With appropriate approval, flyers will also be distributed electronically through community partners and legitimate online platforms. Investigators will engage in additional recruitment at educational and support-focused events for family caregivers, including caregiving walks, conferences, and community events. This may include staffing a vendor table or collaborating with event organizers to distribute study recruitment materials through event newsletters, programs, and digital channels. AANHPI cancer caregivers will also be recruited by leveraging the Collaborative Approach for AANHPI Research and Education (CARE) Registry. The CARE Registry maintains a HIPAA-compliant, institutional review board (IRB)–approved database of AANHPI individuals residing in the United States and US-Affiliated Pacific Islands who have expressed willingness to participate in health-related research. As an established research platform dedicated to reducing disparities in health care studies, CARE provides access to a diverse pool of potential participants. Partnering with this platform allows for a more efficient and targeted recruitment process, ensuring that the study reaches an underrepresented population while maintaining ethical and regulatory compliance. The contact information of the principal investigator, as well as the IRB approval statement and the contact information for the IRB of record, will be included in the informed consent materials of all the electronic surveys and qualitative interviews. Recruitment will span approximately 12-16 months to maximize sample size and ensure sufficient diversity in participant representation.

#### Enrollment and Participation

Interested individuals who meet initial eligibility criteria will be contacted by study staff to schedule an initial phone screening, after which informed consent will be obtained and baseline measures collected through a pretest electronic survey. Participants will be asked to use the eCARE app weekly for 6 sessions (T1-T6) over an 8-week period. Weekly phone-based reminders will prompt engagement with the platform. If an assessment is missed, the app will automatically issue 2 follow-up notifications within 24-48 hours. Platform usage data will be collected to assess adherence (eg, frequency and duration of app use). A brief posttest assessment (T7) will be conducted approximately 8 weeks after baseline; the survey will include the same instruments presented during the pretest assessment plus the acceptability (AIM questionnaire [76]) and user engagement questionnaires (User-Engagement Scale-Short Form [77]).

#### Qualitative Component

A purposive subsample of participants (n=20; 5 per racial and ethnic group) will be invited to complete a semi-structured qualitative interview exploring facilitators and barriers to eCARE uptake, in addition to recommended improvements and refinement. Interest in participating in this component of the project will be assessed during the phone screening and pretest survey outlined in Phase 1. Eligible family caregivers will be contacted at the end of the intervention by email and those who agree will be invited to complete a 60- to 90-minute Zoom (Zoom Video Communications, Inc) interview with a member of the research team. Serial recruitment will continue until thematic saturation is achieved, defined as no new codes or themes emerging in at least 2 consecutive interviews. This approach follows empirical and methodological recommendations, as many core themes in relatively homogenous qualitative samples are typically identified within the first 12 interviews [78-80] and sample sizes of 12-20 are generally sufficient for achieving saturation in focused qualitative studies [81-83]. By exceeding the standard threshold for saturation, the study’s target sample size allows for a more comprehensive exploration of a diverse group of users’ perspectives. Details regarding assessment points and measures are summarized in Table 1.

#### eCARE Intervention

eCARE is an evidence-based artificial intelligence (AI)–enabled D&A screening and monitoring tool using speech data, which analyzes 90 seconds of participant “conversation” on selected topics. After logging in and hitting “start,” participants can “talk to” the app about their mental health by selecting among 3 different prompts. After each recording, users will see a numeric score and a visual interpretation of their results. Then, they will be taken to the next page, where PHQ-9 and GAD-7 will be collected. Caregivers are also presented with mental health and suicidality resources on the last screen of the session.

#### Participant Safety

An individual safety plan will be implemented for family caregivers of individuals diagnosed with cancer endorsing the suicide item of the PHQ-9 and for those who score above the cutoff scores for anxiety and depression on the selected instruments. In case of elevated distress registered during the use of the eCARE app, as evidenced by scores ≥10 on the GAD-7 and the PHQ-9 instruments, participants will have access to mental health resources both in the app and via separate email within 24 hours. For those endorsing the suicide risk item, the eCARE app provides immediate information to the 988 Suicide and Crisis Lifeline, with the contact number for the helpline and chat option visible at the end of the session. The Suicide & Crisis Helpline offers 24×7 call, text, and chat access to trained crisis counselors who can help people experiencing suicidal ideation, substance use, and/or mental health crisis, or any other kind of emotional distress.

In case of high distress, participants will receive an email about mental health resources. In the case of participants who endorse the suicidality item, the email will contain ad hoc resources such as 988 Suicide & Crisis Lifeline, 211, National Alliance on Mental Illness (NAMI) phone and chat option, as well as 911. These communications will be accompanied by a text message in the app as well to alert the participant of the elevated score and that resources have been shared. Participants can also contact the investigators or let the study team members know about their interest in being referred to these community-based organizations. The online eCARE platform will be monitored weekly; however, the system alerts the investigative team in real time for each missed assessment or for assessments where the suicide risk item is endorsed. Importantly, the eCARE application does not interface with other smartphone applications or services (eg, Apple Health or Android Health) and does not collect or transmit protected health information beyond what is consented to within the study protocol.

#### Study End Points

The primary feasibility end point will be determined by the number of times each participant completes the eCARE assessment out of a total of 6 possible time points over an 8-week period. Participants who complete the assessment at least 4 times (≥ 66.6% completion rate) will be considered to have met the feasibility threshold. The feasibility study will be deemed successful if at least 60% of enrolled participants (n≥36) reach this threshold. The secondary acceptability end point will be assessed using the AIM, a psychometrically validated implementation science instrument designed to evaluate participants’ perceptions of intervention acceptability. An average AIM score of 4 out of 5 will be interpreted as indicative of acceptable intervention uptake.

### Measures

As part of this study, we are collecting a comprehensive set of variables to capture the multifaceted experiences of caregiving.

#### Sociodemographic Characteristics

Sociodemographic characteristics include participants’ age, sex, gender identity, race and ethnicity, educational attainment, relationship status, employment status, insurance coverage, and household income.

#### Clinical Characteristics (Care Recipient)

Clinical characteristics of the care recipient—such as their age, sex, cancer diagnosis, cancer stage, and type of treatment—are recorded to contextualize the caregiving experience.

#### Caregiving Characteristics

To further understand the caregiving context, we will gather data on the length of the caregiving period, the caregiver’s relationship to the care recipient, primary caregiver status, and cohabitation. We will also assess caregiving intensity through the number of hours of care provided per week, the types of tasks performed, and the care recipient’s level of independence in activities of daily living, measured by the Katz Index [84].

### Outcome Measures

#### Psychological Distress

Depression is measured by the PHQ-9*,* a validated, self-administered instrument widely used to screen for, diagnose, and monitor the severity of depressive symptoms [64]. Respondents rate the frequency with which they have experienced each symptom over the past 2 weeks on a 4-point Likert scale, ranging from 0 (“Not at all”) to 3 (“Nearly every day”), yielding a total score between 0 and 27. Interpretation of PHQ-9 scores follows standard clinical guidelines: 0-4 (minimal depression), 5-9 (mild), 10-14 (moderate), 15-19 (moderately severe), and 20-27 (severe). A total score of 10 or higher is commonly used as a cutoff for identifying clinically significant depressive symptoms. Item 9 specifically assesses suicidal ideation and requires immediate clinical attention if endorsed. The PHQ-9 has demonstrated strong reliability and construct validity across diverse populations and care settings, including oncology. The excellent psychometric property of the PHQ-9 has been reported across different age and racial or ethnic groups, endorsing its utility among patients with cancer [85]. The GAD-7 is a widely used, self-administered instrument designed to assess the severity of generalized anxiety symptoms over the past 2 weeks. It includes 7 items corresponding to core symptoms of generalized anxiety disorder. Each item is rated on a 4-point Likert scale ranging from 0 (“Not at all”) to 3 (“Nearly every day”), yielding a total score between 0 and 21 [65]. Interpretation of total scores follows established clinical cut points: 0-4 (minimal anxiety), 5-9 (mild), 10-14 (moderate), and 15-21 (severe). A score of 10 or above is commonly used as a threshold for identifying clinically significant anxiety symptoms, warranting further assessment. The GAD-7 demonstrated excellent internal consistency (α=.92), strong test-retest reliability, good criterion, construct, and convergent validity [65,86]. These strong psychometric properties have been confirmed across age groups and racial and ethnic backgrounds, including those diagnosed with cancer [87-89].

#### Closeness

The Inclusion of Other in the Self (IOS) Scale [68,90] is a widely used, single-item pictorial measure of interpersonal closeness. Grounded in self-expansion theory [91,92], the IOS assesses the degree to which individuals perceive their relationship partner as integrated into their sense of self. In the current study, the IOS was used to evaluate perceived emotional closeness between family cancer caregivers and their care recipients. Participants are shown seven pairs of circles labeled “Self” and “Other,” ranging from no overlap (1) to almost complete overlap (7). They are asked to select the pair that best represents their relationship with the care recipient. This visual analog approach provides a rapid and intuitive assessment of relational closeness and has demonstrated validity across diverse populations [69,91,93,94]. The IOS has been linked to a variety of relationship and health outcomes and is commonly used in studies of romantic, familial, and community ties [92]. In this study, caregiver-reported IOS scores provide a snapshot of the emotional bond between caregiver and care recipient, an interpersonal factor that may influence psychological distress, motivation to engage in support tools, and the caregiving experience more broadly.

#### Caregiving Burden

Caregiving burden is assessed with the Zarit Burden Interview-Short Form (ZBI-12) a 12-item self-report measure used to assess the subjective burden experienced by informal caregivers [67]. It evaluates key dimensions of caregiver strain, including emotional, physical, and social impacts associated with caregiving responsibilities. Each item is rated on a 5-point Likert scale ranging from 0 (“Never”) to 4 (“Nearly always”), yielding a total score ranging from 0 to 48, with higher scores indicating greater perceived burden. Standard scoring guidelines categorize burden levels as follows: 0-10 (no to mild burden); 10-20 (mild to moderate burden); >20 (high burden). The ZBI-12 has demonstrated strong reliability and validity across diverse caregiving populations, including those caring for individuals with dementia, cancer, and other serious illnesses.

#### Communication

The Social Constraints Scale (SCS) is a 15-item self-report instrument developed to assess the extent to which individuals perceive their social environment, particularly close others such as spouses or family members, as inhibiting or discouraging the expression of illness-related thoughts and emotions [70,71]. In the context of cancer, the SCS captures perceived social responses that constrain open communication, such as minimizing concerns, changing the subject, or expressing discomfort when the patient discusses their cancer-related experiences. Each item is rated on a 4-point Likert scale ranging from 1 (“Never”) to 4 (“Often”), with higher total scores (ranging from 15 to 60), indicating greater perceived social constraint. The SCS has demonstrated strong psychometric properties across diverse populations coping with chronic illness.

#### Perceived Responsiveness

Caregivers’ perceptions of care recipient responsiveness were measured by the 12-item Perceived Partner Responsiveness Scale (PPRS) [72]. The PPRS measures the degree to which people feel their loved ones are responsive to them. It measures two dimensions of perceived responsiveness in close relationships: (1) understanding (7 items, eg, “My loved one gets the facts right about me;” “My loved one knows me well”) and (2) validation (5 items, eg, “My loved one values and respects the whole package that is the real me;” “My loved one seems interested in what I am thinking and feeling”). Responses were scored on a 9-point Likert scale ranging from 1 (not at all true) to 9 (completely true). A total score is calculated by summing all items [72], with scores indicating greater perceptions of others’ responsiveness.

#### Communal Motivation to Care

Communal motivation was assessed with the 10-item Partner-Specific Communal Motivation Scale [74]. Caregivers were asked to assess to what extent they were communally motivated to care for the well-being of their care recipients. Responses were scored on a 9-point Likert scale ranging from 1 (not at all) to 9 (extremely). Example items are “Helping my loved one is a high priority for me,” “I would sacrifice very much to help my loved one,” “I would be reluctant to sacrifice for my loved one*.*” The total score is given by the sum of the items after reversing items 2, 5, and 10, and indicates higher levels of communal motivation.

#### Quality of Life

The Centers for Disease Control and Prevention (CDC) healthy days measure, commonly referred to as health-related quality of life-4 (HRQOL-4), is a brief, validated instrument designed to assess health-related quality of life in population-based studies and public health surveillance [66]. The HRQOL-4 includes 4 core items, including self-rated general health, physically unhealthy days, mentally unhealthy days, and physical or mental health interference with daily activities. Each item captures a distinct domain of the HRQOL-4, allowing for both individual and composite assessments of overall well-being. The HRQOL-4 has demonstrated strong reliability and validity across diverse populations [66,95].

#### Acceptability of Intervention

The AIM is a pragmatic, 4-item self-report instrument developed to assess stakeholders’ perceptions of the acceptability of a specific intervention or implementation strategy [76]. The AIM captures the extent to which an intervention is perceived as agreeable, satisfactory, or palatable, with items rated on a 5-point Likert scale ranging from “completely disagree” to “completely agree.” The items include (1) “(Intervention) meets my approval,” (2) “(Intervention) is appealing to me,” (3) “I like (Intervention),” and (4) “I welcome (Intervention).” It has been designed for use across diverse stakeholder groups, including service providers, administrators, and caregivers, and it requires no specialized training for administration or interpretation. While no established cutoff scores exist, higher total scores indicate greater acceptability. The AIM has demonstrated strong psychometric properties, including content and structural validity, reliability, and responsiveness to change [76].

#### User Engagement

The User Engagement Scale (UES-SF) is a 12-item self-report instrument developed to assess user engagement in human-computer interaction contexts [77]. The short form includes four key subscales, each with 3 items: focused attention, perceived usability, aesthetic appeal, and reward*.* Participants respond using a 5-point Likert scale ranging from 1 (“Strongly Disagree”) to 5 (“Strongly Agree”). Per scoring instructions, items within the perceived usability subscale are reverse coded. Subscale scores are computed by averaging the responses within each domain, and a total engagement score is calculated by averaging across all 12 items. The UES-SF was designed for high usability, with items written at a fifth-grade reading level and no special training required for administration or interpretation. The instrument has demonstrated sound psychometric properties, including content and structural validity, internal consistency, and responsiveness to user experiences [77].

### Data Analysis

Quantitative and qualitative data will be analyzed using a mixed methods approach to evaluate the feasibility and acceptability of the eCARE tool among family caregivers of individuals diagnosed with cancer. Descriptive statistics will be used to summarize participants’ sociodemographic, clinical, and caregiving characteristics, including means, SDs, ranges, and 95% CIs. Graphical methods (eg, histograms, boxplots, and scatterplots) will be used to examine variable distributions and identify the need for transformations. For comparisons, we will use ANOVA for continuous variables and chi-square tests for categorical variables. Differences in psychological distress, caregiving burden, relational closeness, perceived responsiveness, and motivation to provide care will also be explored. Feasibility, the primary outcome of Aim 1, will be assessed based on the number of participants completing at least 4 of 6 total weekly eCARE assessments during the 8-week study period. Participants exceeding 6 sessions due to more frequent use will be classified as having met the feasibility threshold, consistent with those completing at least 4 of 6 sessions. The study will be considered feasible if 60% or more of enrolled participants (≥36 of 60) achieve this end point. Acceptability, the secondary outcome, will be assessed using the AIM [76]. The mean and SD of AIM scores will be calculated, with an average score of ≥4.0 (on a 5-point scale) interpreted as evidence of acceptability. Exploratory latent growth curve modeling will be used to examine changes in depression and anxiety symptoms over time (measured by PHQ-9 and GAD-7). These analyses are considered exploratory due to the limited statistical power associated with the sample size (n=60).

For Aim 2, qualitative data from semi-structured interviews with a purposive subsample of 20 family caregivers (including both completers and noncompleters) will be analyzed using thematic content analysis in ATLAS.ti [96]. Interview transcripts will be transcribed verbatim and independently coded by trained research staff. An initial coding scheme will be developed inductively and applied iteratively. Two independent coders will review all transcripts, with discrepancies resolved through discussion until consensus is achieved [97]. Axial coding and thematic mapping will be used to identify higher-order categories related to eCARE’s perceived strengths and limitations, usability, relevance, and reported facilitators or barriers to engagement. Analysis will continue until thematic saturation is reached, defined as the point at which no new codes or themes emerge [79,97,98].

### Ethical Considerations

This study was reviewed and approved by the IRB at the University of Houston (Protocol# STUDY00003186) and adheres to the ethical standards outlined in the Declaration of Helsinki. All study procedures will be conducted in accordance with the approved protocol and applicable institutional requirements for human subjects’ research.

Participants will provide informed consent prior to initiating any study procedures. Consent will be documented in writing at enrollment either via Qualtrics e-signature functionality or by signing a printed consent form when enrollment occurs in person. For the optional qualitative interview component, participants will complete an additional oral consent procedure prior to the start of the interview, and the study team will request a waiver of written documentation for that interview-specific consent, as applicable.

Participant privacy and confidentiality will be protected through multiple safeguards. Direct identifiers (eg, name, email, phone) will be collected solely for recruitment, scheduling, and compensation purposes and will be stored separately from research data, linked only via a study ID. App-based and survey data will be maintained on encrypted, password-protected platforms with access restricted to authorized study personnel; downloaded data will be stored on password-protected, encrypted university devices. While participants’ interest in the qualitative component will be captured as part of the informed consent process, oral informed consent will be obtained again prior to the commencement of the qualitative interviews. Qualitative interviews will be audio and/or video-recorded only with participant permission; recordings will be transcribed and then deleted following transcription. Interview transcripts will be labeled with study IDs. Only approved members of the research team will have access to identifiable information, and the master linkage file will be maintained by the principal investigator and destroyed at study completion per protocol.

Participants will receive incentives in the form of electronic Amazon gift cards. To minimize the risk of undue inducement, gift cards will be distributed at three predetermined time points, including US $30 upon completion of a baseline consent and pretest assessment survey, US $30 at the midpoint assessment (T4) contingent on at least one completed check-in with eCARE, and US $30 upon completion of the posttest assessment (T7). Participants who complete the qualitative interview will receive an additional US $50 e-gift card.

## Results

Participant recruitment and enrollment began in June 2024, with data collection expected to conclude in August 2025. Data analysis will commence in December 2025, coinciding with the PI’s transition to a new institution, with preliminary findings anticipated by April-May 2026. Table 2 presents the baseline sociodemographic, clinical, and caregiving-related characteristics of enrolled participants, while Table 3 outlines the primary feasibility outcomes and secondary outcomes of the pilot study (Multimedia Appendix 1).

**Table 2:** Baseline characteristics of the participants in the feasibility study.

Category and variable		Measurement
**Sociodemographic characteristics**		
	Age	Years
	Sex	Male, female, or other
	Gender	Self-reported
	Race and ethnicity	US Census categories; self-reported
	Education	Highest level completed
	Relationship status	Married, partnered, single, etc
	Employment	Full-time, part-time, unemployed, or not applicable
	Insurance coverage	Private, public, other or uninsured
	Income	Income brackets
**Clinical characteristics of care recipient**		
	Age	Years
	Patient sex	Male, female, or other
	Cancer diagnosis	As reported by family caregivers
	Cancer treatment	Chemotherapy, radiation, surgery, etc
	Cancer stage	I-IV or not applicable
**Caregiving characteristics**		
	Length of caregiving period	Months or years
	Relationship with care-recipient	Categories based on self-report
	Primary caregiver status	Yes or No
	Cohabitation with care recipient	Yes or No
	Caregiving intensity (hours/week)	Total hours per week
	Type of tasks provided	Household, personal, practical, and emotional
	Care recipient ADL^a^ Independence (Katz Index)	Yes or No; score range 0-6

^a^ADL: activities of daily living.

**Table 3:** Primary and secondary outcomes of the feasibility study.

Outcome type and measure		Definition/Criteria	Success threshold
**Primary outcome**			
	Feasibility end point (eCARE^a^ completion rate)	Number of eCARE completions during 8-week period Proportion of participants achieving feasibility end point	≥4 completions (≥66.6%) = end point achieved Study feasible if ≥60% of participants (≥36) reach this threshold
**Secondary outcome**			
	Acceptability of Intervention Measure (AIM)	Participant-reported acceptability of eCARE using AIM scale (range: 1 to 5)	Average score ≥4 over 5

^a^eCARE: Ellipsis Caregiver Assessment Enhancement.

## Discussion

### Principal Findings

Family caregivers play a central role in cancer care, assuming extensive practical, emotional, and coordination responsibilities that contribute to substantial psychological, physical, and financial strain [1,2,11]. Across cancer types and illness stages, caregivers frequently experience clinically significant anxiety and depression, often at rates exceeding those observed in patients themselves, yet their mental health needs remain insufficiently addressed [4,5,7-9,22]. This single-arm, prospective pilot study is among the first to evaluate the feasibility and acceptability of using artificial intelligence and speech-based analytics through a mobile app (eCARE) to monitor the severity of depression and anxiety among a sample of community-based family caregivers of individuals living with cancer. Traditional methods, such as clinic-based assessments or paper forms, often present challenges with high participant burden and low engagement, which can compromise data accuracy and limit opportunities for timely intervention. It is essential to explore innovative strategies offering more flexible, scalable, and caregiver-centered approaches to distress monitoring. To this end, we hypothesize that (1) eCARE will be feasible and acceptable, and (2) that participants will provide in-depth information related to barriers and facilitators influencing its uptake, in addition to feedback for future refinement and implementation.

Prior research has demonstrated that eHealth interventions for cancer caregivers can reduce depressive symptoms and modestly improve quality of life; however, existing studies are few, heterogeneous, and often limited by short follow-up periods and narrow outcome focus [6,12,34,35,39,99]. Moreover, most digital interventions rely on self-report questionnaires, which can introduce response burden and missing data. By requiring a brief speech sample to generate predictions of depression and anxiety, eCARE represents a minimally burdensome pathway for mental health monitoring. The mobile application integrates natural language processing to analyze spoken words and acoustic modeling to assess vocal features, leveraging an accessible, multimodal approach well-suited for smartphones [37,39,59,99,100]. As Schoenberg [101] highlighted, digital tools that are frictionless and embedded in daily life are more likely to be adopted and sustained in real-world environments.

Findings from this pilot study will provide preliminary data on key indicators of feasibility (eg, completion rates) and acceptability (eg, satisfaction ratings). This information will be instrumental in helping to determine whether a speech-based tool can be successfully integrated into the complex caregiving context, where time constraints, emotional burden, and competing demands often limit participation in mental health initiatives. To further this goal, the study also incorporates qualitative feedback, allowing for a user-informed refinement of the application’s content, usability, and perceived value. While eCARE does not currently involve provider feedback loops, future iterations may benefit from exploring how clinicians can access and respond to data trends emerging from the app, potentially enhancing care coordination and timely referral to supportive services.

Should eCARE demonstrate feasibility and acceptability, these findings will directly inform the design of a larger, multisite randomized controlled trial. Such a trial would be designed to robustly compare eCARE with established screening methods, like the National Comprehensive Cancer Network (NCCN) Distress Thermometer or standard electronic symptom checklists, and to evaluate its efficacy on family caregivers’ mental health.

Finally, since symptoms of depression and anxiety are among the most prominent predictors of quality of life and long-term health outcomes, effective and early identification is critical. If validated, speech-based assessments like eCARE could serve as a foundation for future interventions aimed at improving caregiver resilience, reducing emotional burden, and ultimately enhancing the caregiving experience.

### Limitations

This study has several limitations that should be considered. First, as a single-arm feasibility and acceptability study, it is not designed to evaluate the efficacy of eCARE in reducing depression or anxiety symptoms, nor to support causal inferences. Second, generalizability may be limited, as participation requires access to a smartphone and a minimum level of comfort with mobile technology, potentially excluding family caregivers with lower digital literacy or limited access to digital resources. Third, self-selection bias may be present, as caregivers who choose to enroll may be experiencing higher distress, may be more motivated, or may be more receptive to digital mental health tools, which could inflate estimates of feasibility and acceptability. Finally, the relatively short monitoring period affects the ability to elaborate on sustained engagement and longer-term acceptability, in addition to barriers and facilitators influencing eCARE uptake over time.

### Conclusions

This study will generate foundational evidence on the feasibility, acceptability, and perceived utility of a mobile, speech-based, AI-enabled tool for monitoring depression and anxiety among family caregivers of individuals living with cancer. Findings will inform the design of a fully powered clinical trial and guide the optimization of remote mental health screening approaches tailored to the caregiving experience. As a scalable, low-cost, and low-burden intervention, eCARE has the potential to support timely identification of psychological distress, enhance access to supportive care services, and improve coordination of psychosocial care across oncology settings. Beyond cancer caregiving, the underlying model holds promise for adaptation to other chronic or life-limiting conditions and to non–English-speaking populations, pending appropriate validation. Future studies should compare eCARE with established screening tools and examine long-term clinical outcomes, user engagement, and effects on both caregiver and patient well-being, while also evaluating strategies for integrating AI-derived screening results into clinical workflows without increasing provider burden. Dissemination of findings will include sharing results with academic, clinical, and community stakeholders to support continued refinement and implementation of digital mental health solutions for caregivers.

## Data Availability

The datasets generated or analyzed during this study will be available from the corresponding author on reasonable request.

## Appendix

Multimedia Appendix 1 CONSORT checklist.

Multimedia Appendix 2 Peer-review report from the University of Houston SEED Grant Review Committee.

## Abbreviations

AI: artificial intelligence
AIM: acceptability of intervention measure
AANHPI: Asian American, Native Hawaiian, and Pacific Islander
CARE: Collaborative Approach for AANHPI Research and Education
CDC: Centers for Disease Control and Prevention
D&A: Depression and Anxiety
eCARE: Ellipsis Caregiver Assessment Enhancement
GAD-7: Generalized Anxiety Disorder–7
HIPAA: Health Insurance Portability and Accountability Act
HRQOL-4: Health-Related Quality of Life – 4 item Healthy Days Measure
IOS: Inclusion of the Other in the Self Scale
IRB: Institutional Review Board
NAMI: National Alliance on Mental Illness
NCCN: National Comprehensive Cancer Network
PHQ-9: Patient Health Questionnaire–9
PPRS: Perceived Partner Responsiveness Scale
SCS: Social Constraints Scale
UES-SF: User Engagement Scale–Short Form
ZBI-12: Zarit Burden Interview–Short Form (12 items)
