# Supplementary material for: Evaluating an AI-Enabled Mobile Mental Health Monitoring Tool Among Family Caregivers of Adults Living With Cancer: Single-Arm Feasibility and Acceptability Trial Protocol
Source: JMIR Res Protoc. 2026 Feb 12;15:e83276. doi: 10.2196/83276 (PMC12946784; doi:10.2196/83276)
Supplement: Multimedia Appendix 2 [file resprot_v15i1e83276_app2.pdf]

Division of Research
UNIVERSITY OF HOUSTON

May 5, 2023

Chiara Acquati, PhD
Graduate College of Social Work

Re: 2022-2023 High Priority Area Research Seed Grants

Dear Dr. Acquati,

The Research and Scholarship Committee has completed the review process for the High Priority Area Research Seed Grants. Your application was reviewed by the selection committee, which consisted of RSC members and members recruited from across the campus with expertise in one or more of the five high priority areas (see guidelines). The overall impact score ranges on a scale from 1 (highest) to 5 (lowest). While this takes the five criterion scores into consideration it is also driven by the likelihood your proposal will result in a fundable application. Your application received an average overall impact score of **2.10**. For your reference, we received 26 applications and the scores ranged from 1.5 to 4.25.

Congratulations! Your request for support has been approved in the amount of **$70,000**.

According to University Policy, your project will undergo a congruency review by the Research Integrity and Oversight office before a cost center can be established. If your research involves Human Subjects, Animals, Biohazards, and/or Radiation, you will need to obtain the corresponding approvals, and the proposal will need to be congruent with the approved protocols. *This review must be completed within 90 days of the date on this letter or your award will be forfeited. In addition, only one 6-month extension will be provided except for clearly observed obstacles, such as a need for parental leave. Do not accept this award if your timetable for project completion and grant submission is unrealistic.*

Please note the required deliverables and ensure that your progress reports and grant submissions occur in accordance with the requirements of the award: The PI is expected to complete the following activities and reporting during the 2 years following the award. An external grant proposal must be submitted in one of the high priority research areas in Year 1 of the project period. If the applicant intends to apply for a competition that does not fit this timeline, an exception should be noted in the proposed timeline. An exception can be granted by petition provided this extension delays the grant submission for no more than 3 months. A resubmission is expected in the next submission cycle following the receipt of reviewer comments until funding is secured or a new application can be submitted while awaiting the outcome of the previous submission. In Year 2, the PI is expected to submit either a second proposal or resubmit the proposal from Year 1. At the end of the funding period, there will be a final report that captures the research output and funding garnered using this grant. Any investigators who do not submit timely progress reports or show adequate progress will have their funding terminated. It is the responsibility of each award recipient to provide information to the Division of Research concerning all external grant applications that are submitted or awards received as a result of this funding. Such information should include the date of submission, the title of the project, inclusive dates, agency, total amount requested, and the status of each application. Failure to comply with this reporting requirement will disqualify an individual for future consideration in all internal funding programs sponsored by the Division of Research. Notice must be given of publications or presentations resulting from the award. The grantee must acknowledge University support in all publications or presentations resulting from the award and must provide one copy of the publication to the Division of Research, mail code 2015.

Division of Research
UNIVERSITY OF HOUSTON

Progress reports must be submitted using the Progress Report link on the Internal Awards webpage on their due dates: May 14, 2023 —funding available; reports due November 12, 2023, May 13, 2024, with final report November 11, 2024. Funds expiration: November 1, 2024.

All questions related to this program should be submitted to Dr. Ezemenari Obasi, Associate Vice President for Research Administration, emobasi@uh.edu.

Sincerely,

Claudia Neuhauser, PhD
Interim Vice Chancellor/Vice President for Research

Division of Research
UNIVERSITY OF HOUSTON

2023 SEED Proposal Scores and Reviewer Comments

PI: Chiara Acquati

Proposal Title: Evaluating the Feasibility and Acceptability of a Mobile Artificial Intelligence (AI)-enabled Depression and Anxiety Monitoring Tool among Racial and Ethnic Minority Cancer Caregivers

Review Criteria: The *High Priority Area Seed Grant (SEED) program* seeks to provide funds that would permit submission of competitive research proposals. Preference is given to bold new ideas showing clear evidence of leading to additional grant support.

1. Short-term impact of proposed research
2. Quality of the research methods
3. Investigator expertise and track record
4. Plan for grant submission
5. Long-term potential for substantive contributions to research area

Review Comments:

Strengths: This study aims to develop a modeling approach that incorporates deep learning, satellite-based remote sensing, and numerical modeling techniques to produce gap-free grid-based estimates of air pollutant concentrations over the U.S. from 2012 to 2021. Creatively examines long-term variations in air quality and assess pollutant source attributions across nine distinct climate regions. The main innovations are to improve their existing DNN model with a new dataset and use gap-free observation references obtained to enable long-term source apportionments for the various pollutants. Team covers all bases and has past research on both deep learning and modeling and are qualified to conduct the proposed research. Proposal clarifies the gap in the research and explains how and why Black and Hispanic caregivers' experiences differ from their White counterparts. The aims of the research are appropriate for a SEED grant and pave the way for further funding, should the researchers find that the intervention is feasible and acceptable. The expected outcomes include a specific NIH grant opportunity. The team is uniquely qualified to conduct the research described. The budget is appropriate. Research methods, focusing on a critical issue. This project proposes to test the feasibility and acceptability of an artificial intelligence (AI) enabled mental health assessment tool among cancer caregivers from minority groups, aiming to address their depression and anxiety. The team is well qualified to conduct the proposed activity.
Weaknesses: The authors present different agencies to which they might submit grants. National Cancer Institute application targeting the R01 grant mechanism (PAR-22-145) "Leveraging Health Information Technology to Address and Reduce Health Care Disparities." However, there is no plan for external grant submission in year 1 as required in the guidelines (reporting and acknowledgement section) or for grant revision (grant submission proposed only in year 2). Details on assuring data integrity or general QA/QC absent. The results would be specific to the use of ECare which is a proprietary product and results may not be generalizable to other tools. The proposal should explain what data exist (if any) on the feasibility and acceptability of the intervention, and why those data (if they exist) are insufficient. Why are the preliminary data reported insufficient? The timeline includes a lot of data collection, some of which overlaps with dissemination and grant proposal write-up. I wonder how feasible it is to have so much of the work overlap. The RA salary is lower than what GCSW normally pays. novelty; What is new, original; whats a significant addition to body of knowledge. Psychology is totally out of the reviewer's expertise, so it is really hard for the reviewer to judge the contribution of this proposal. However, the approach of this proposal is like trial of a digital

tool, collecting data, and conducting data analysis, which is typical for social science research. By saying that, the problem for us is are we funding the problem or funding the technique? If the former, this project should be funded, since the problem is important; if latter, the data collection procedure and analysis approach is nothing special.

Overall Impact Scores ranged from 1 (Highest) to 5 (Lowest):

Reviewer 1: 2

Reviewer 2: 1.3

Reviewer 3: 3

Reviewer 4: 2

Ezekiel W. Cullen Building • 4302 University Drive, Room 316 • Houston, TX 77204-2015
Office: 713.743.9104 • Fax: 713.743.9577 • www.uh.edu/research
